# Inhibition of autophagy in EBV-positive Burkitt's lymphoma cells enhances EBV lytic genes expression and replication

**DOI:** 10.1038/cddis.2015.156

**Published:** 2015-09-03

**Authors:** A De Leo, F Colavita, F Ciccosanti, G M Fimia, P M Lieberman, E Mattia

**Affiliations:** 1 Department of Public Health and Infectious Diseases, Sapienza University of Rome, 00185 Rome, Italy; 2 National Institute for Infectious Diseases “Lazzaro Spallanzani” IRCCS, 00149 Rome, Italy; 3 Department of Biological and Environmental Sciences and Technologies (DiSTeBA), University of Salento, 73100 Lecce, Italy; 4 The Wistar Institute, Philadelphia, PA, USA

## Abstract

Autophagy, an important degradation system involved in maintaining cellular homeostasis, serves also to eliminate pathogens and process their fragments for presentation to the immune system. Several viruses have been shown to interact with the host autophagic machinery to suppress or make use of this cellular catabolic pathway to enhance their survival and replication. Epstein Barr virus (EBV) is a *γ*-herpes virus associated with a number of malignancies of epithelial and lymphoid origin in which establishes a predominantly latent infection. Latent EBV can periodically reactivate to produce infectious particles that allow the virus to spread and can lead to the death of the infected cell. In this study, we analyzed the relationship between autophagy and EBV reactivation in Burkitt's lymphoma cells. By monitoring autophagy markers and EBV lytic genes expression, we demonstrate that autophagy is enhanced in the early phases of EBV lytic activation but decreases thereafter concomitantly with increased levels of EBV lytic proteins. In a cell line defective for late antigens expression, we found an inverse correlation between EBV early antigens expression and autophagosomes formation, suggesting that early after activation, the virus is able to suppress autophagy. We report here for the first time that inhibition of autophagy by Bafilomycin A1 or shRNA knockdown of Beclin1 gene, highly incremented EBV lytic genes expression as well as intracellular viral DNA and viral progeny yield. Taken together, these findings indicate that EBV activation induces the autophagic response, which is soon inhibited by the expression of EBV early lytic products. Moreover, our findings open the possibility that pharmacological inhibitors of autophagy may be used to enhance oncolytic viral therapy of EBV-related lymphomas.

Epstein Barr Virus (EBV) causes infectious mononucleosis and it is associated with several tumors of both epithelial and B-cell origin such as Burkitt's lymphoma (BL), nasopharyngeal carcinoma, Hodgkin's disease and lymphoproliferative disorders occurring in post-transplant patients and human immunodeficiency virus (HIV)-infected individuals. Alike all herpes viruses, EBV has both a latent and a lytic replication program. Besides the oncogenic potential of several EBV latent genes, lytic products may also be relevant for the development of EBV-associated malignancies.^1^ EBV productive infection, allowing the virus to spread, increases the pool of latently infected cells increasing the risk of a clonal expansion of EBV-positive B lymphocytes in immunocompromised individuals. Mechanisms regulating the switch from latent to lytic infection may enable new strategies for treating EBV-associated cancers, referred to as oncolytic therapy.^2, 3^ Physiological stimuli that trigger viral reactivation *in vivo* have not been clearly identified. However, *in vitro,* viral reactivation can be achieved by treating EBV latently infected cells with different chemical or biological inducers, such as 12-O-tetradecanoylphorbol-13-acetate, calcium ionophore, histone deacetylase (HDAC) inhibitor, anti-immunoglobulin G (anti-IgG, IgG) or transforming growth factor beta (TGF*β*).^4^ During the lytic cycle, the sequential expression of immediate early, early and late genes leads to viral particles production. The two immediate early products, BZLF1 and BRLF1, promote each other expression, transactivate separate classes of EBV lytic genes and together synergize to activate a third class of lytic genes.

Several studies indicate that autophagy has a role in pathogen infection. Autophagy is a complex degradation process by which cytoplasmic material, including soluble macromolecules and organelles, is delivered to lysosomes. Autophagy-related genes (ATG) have been identified in yeast, and many of them have orthologs in mammalian cells.^5^ These genes control a multistep mechanism leading to the formation of the autophagosome, a double-membrane vesicle, which, by fusing with lysosomes, forms the autolysosomes where the delivered contents are ultimately degraded. Under normal conditions, the serine/threonine kinase mammalian target of rapamycin (mTOR) phosphorylates Ulk-like kinase 1 and 2, thus repressing autophagy induction.^6^ Conditions such as starvation, metabolic stress, response to some microbial infection or treatment with mTOR inhibitor rapamycin impede mTOR kinase activity, thus activating autophagy. Initially, Beclin1 (BECN1), which constitutively binds cellular Bcl2, forms a complex with Vps34, a phosphatidylinositol 3-kinase (PI3K), which participates in autophagosome nucleation.^7^ During the elongation step of autophagy, microtubule-associated protein light chain 3-I (LC3-I) is processed and conjugated with phosphatidylethanolamine as LC3-II form. Lipidated LC3-II associates with autophagosomal membrane and therefore serves as a marker for autophagosome formation.^8^

Autophagy can function as an antimicrobial defense by contributing to degrade intracellular microorganisms, and to regulate innate and adaptive immunity against viral infections. Some viruses, such as herpes simplex virus type 1 (HSV1), human cytomegalovirus (HCMV), Kaposi's sarcoma-associated herpesvirus (KSHV) and HIV have evolved molecular mechanisms to successfully evade autophagy.^9, 10, 11, 12, 13^ Moreover, other viruses such as coxsackie virus, dengue virus, influenza A virus, hepatitis C virus, varicella-zoster virus and BK polyomavirus induce and profit from autophagy to promote their own replication.^14, 15, 16, 17, 18, 19^

In this study, we investigated the role of autophagy during EBV productive infection in cellular models allowing the complete or partial expression of the viral genome. We evaluated the expression of key autophagy proteins and the formation of autophagic vesicles in BL cells exposed to physiologically relevant EBV activating compounds such as anti-IgG and TGF*β* or to chemical inducers. Moreover, by altering the pathway with autophagy modulators and shRNA-based suppression of Beclin1 expression, we found that inhibition of autophagy in the early as well as in the late phase of the process, largely promotes EBV transcription and replication. We suggest that the host cell enhances autophagy as a response to viral reactivation, but early in the lytic cycle of infection, EBV is able to counteract autophagy.

## Results

### Induction of EBV lytic cycle transiently activates autophagy

**Figure 1:**
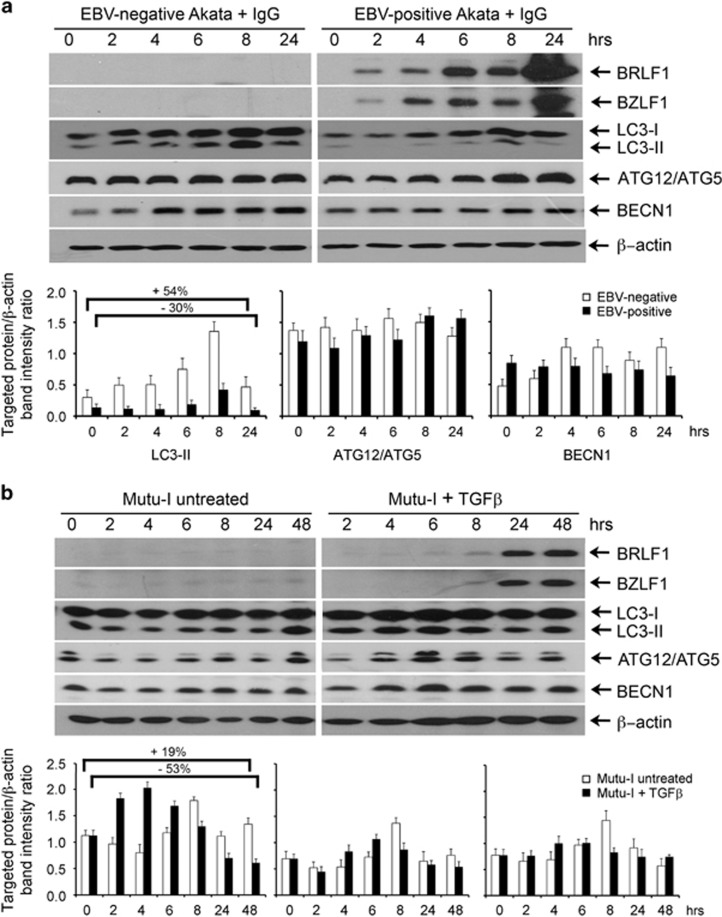
Expression of autophagy marker proteins in EBV-infected BL cells. (**a**) EBV-negative and EBV-positive Akata cells were treated with IgG as described in the Materials and Methods section. Cell extracts from samples collected at the indicated times were analyzed by immunoblotting using antibodies against BRLF1, BZLF1, LC3, ATG12/ATG5, BECN1 and *β*-actin as loading control. (**b**) Mutu-I cells untreated or treated with TGF*β* were analyzed as in **a**. The relative levels of the targeted proteins were obtained by densitometric analysis of the ratio of the specific signals to *β*-actin. The data represent the mean±S.D. of three independent experiments

In isogenic EBV-negative and EBV-positive Akata cells exposed to anti-IgG and Mutu-I cells treated or not with TGF*β*, we measured the levels of three autophagy marker proteins. By immunoblot analysis, we examined the expression of LC3-II that correlates with the formation of the autophagosomes; ATG12/ATG5 as autophagy conjugation system and Beclin1 involved in the early steps of the autophagy pathway. Figure 1a shows that LC3-I and LC3-II components were more abundant in the cells not infected by EBV. LC3-II levels in EBV-negative Akata cells markedly increased during the first 8-h exposure to IgG but decreased thereafter. Treatment of EBV-positive Akata cells with IgG for the same lengths of time induced the expression of BZLF1 and BRLF1 indicating the extent of EBV lytic cycle activation. LC3-II modulation in these cells showed a trend similar to that observed in EBV-negative Akata cells. However, the relative increment of LC3-II between 6 and 8 h, and the following reduction between 8 and 24 h were higher (2.3- and 4.3-fold) in the EBV-positive as compared with the EBV-negative cells (1.9- and 3-fold). Moreover, in the samples of EBV-positive Akata cells collected at 24 h, LC3-II was 30% less than the amount measured at time zero. In contrast, in the EBV-negative cells, at 24 h LC3-II, signal was ~50% higher than that measured at time zero. Neither ATG12/ATG5 complex nor Beclin1 levels changed significantly in EBV-positive and EBV-negative cells. Figure 1b shows the results obtained from Mutu-I cells untreated or exposed to TGF*β* to induce EBV lytic cycle. EBV lytic transactivators BZLF1 and BRLF1 were more strongly detected in these cells after 24 h exposure to TGF*β*. About a twofold increment of LC3-II occurred during the first 4 h of incubation followed by a drastic decrement. In the 24- and 48-h samples, LC3-II content was ~50% of that detected at time zero. Smaller variations were measured in the levels of ATG12/ATG5 complex and Beclin1, with a pattern similar to that of LC3-II. Changes in the marker levels also occurred in untreated Mutu-I cells incubated under the same conditions used for EBV activation by TGF*β*.

To confirm autophagic flux with EBV activation, the levels of LC3-II were determined following treatment with or without Bafilomycin A1, an inhibitor of lysosomal degradation.

**Figure 2:**
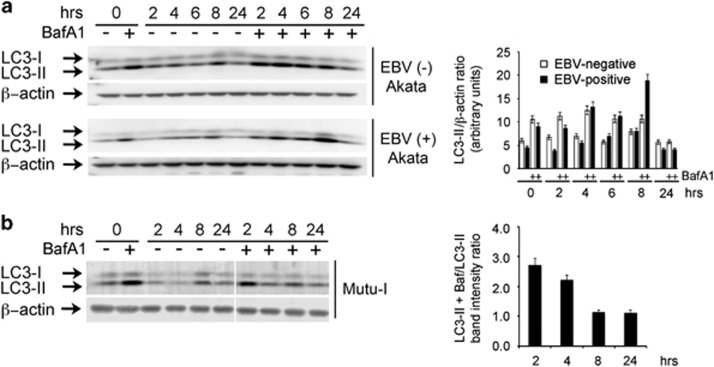
LC3-II turnover assay. EBV- positive and EBV-negative Akata cells (**a**) and Mutu-I cells (**b**) were treated with IgG or TGF*β*, respectively, in the absence or in the presence of Bafilomycin A1 (BafA1) as described in the Materials and Methods section. At the indicated times, samples were subjected to immunoblot analysis with LC3 antibodies. The bar graph in **a** illustrates the densitometric measurements of the LC3-II signals normalized to *β*-actin. The values of the bar graph in **b** represent the ratio between the densitometric measurements of the LC3-II signals detected in the presence and in the absence of BafA1, normalized to *β*-actin. The data represent the mean±S.D. of three independent experiments

As shown in Figure 2a, addition of IgG activates autophagy both in EBV-negative and EBV-positive Akata cells. However, as compared with the uninfected ones, EBV-positive Akata cells showed a substantial increment of LC3-II after 8 h incubation with the inhibitor. In addition, independently of the treatment with Bafilomycin A, similar levels of LC3-II were detected in the samples collected at 24 h, suggesting inhibition of the autophagic flux. These results indicated that the increment of the LC3-II component in EBV-positive Akata cells depended not only on the cellular response to IgG exposure but also to a transient stimulation of autophagy that correlated with EBV reactivation. Similarly, Mutu-I cells were incubated with TGF*β* in the absence and presence of Bafilomycin A1. The bar graph of Figure 2b clearly indicates an increment of the autophagic flux in the first 4 h of incubation while no differences were measured at later time points, thereby suggesting an arrest of the autophagic flux also in this cell line along with EBV reactivation.

### Modulation of autophagy activity affects EBV lytic antigens expression

**Figure 3:**
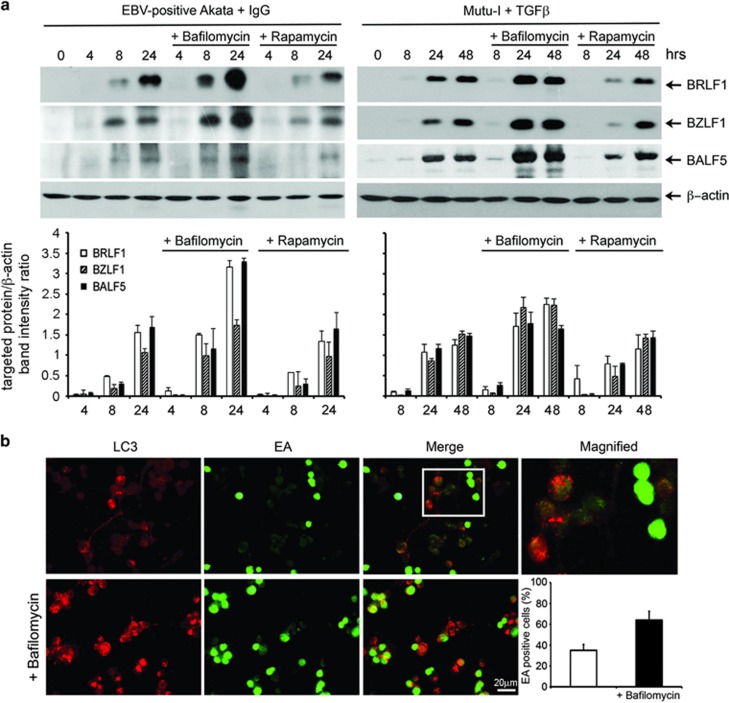
Effects of autophagy modulators on EBV lytic antigen expression. (**a**) EBV lytic cycle was induced in Akata and Mutu-I cells with IgG and TGF*β*, respectively, in the absence or in the presence of either Bafilomycin A1 or Rapamycin. Lysates obtained from the cell samples collected at the indicated times were subjected to immunoblot analysis using antibodies against BRLF1, BZLF1 and BALF5. *β*-Actin was used as loading control. A representative experiment out of three is shown. The relative levels of the targeted proteins were estimated by densitometry and the ratios were calculated relative to *β*-actin. The data represent the mean±S.D. of three independent experiments. (**b**) Raji cells treated with EBV inducing agents for 48 h in the absence or in the presence of Bafilomycin A1 were fixed and stained with anti-LC3 and anti-EA antibodies, followed by the corresponding secondary antibodies conjugated to TRITC and FITC, respectively, as described in the Materials and Methods section. The magnified image corresponds to the white-square frame-enclosed region enlightened in the merge image. Scale bar, 20 *μ*m. The bar graph illustrates the data as the percentages of EA-positive cells in the absence or in the presence of Bafilomycin A1. The data represent the mean±S.D. of three similar experiments

To explore the relationship between autophagy and EBV lytic infection, the virus productive cycle was induced in Akata and Mutu-I cells in the absence or in the presence of pharmacological agents that either inhibit or stimulate the autophagic pathway. Bafilomycin A1, a vacuolar ATPase inhibitor that blocks lysosomal acidification as well as the fusion of autophagosomes with the lysosomes, and Rapamycin, a drug that inhibits mTOR, were used to inhibit or to enhance autophagy, respectively. Cell samples collected at 8 and 24 h were analyzed by immunoblot for the expression of EBV lytic antigens BZLF1, BRLF1 and the early DNA polymerase BALF5. The activity of the two autophagy modulators was verified by detecting the increment of LC3-II with Bafilomycin A1 and the reduced phosphorylation of the TORC1 substrate p70S6 with Rapamycin (data not shown). Figure 3a shows that exposure of Akata and Mutu-I cells to EBV activators in the presence of Bafilomycin A1 strongly increased lytic antigens expression. In contrast, Rapamycin slightly affected viral protein levels in both cell lines, suggesting that the increment of autophagy by EBV activation does not involve mTOR signaling.

It was reported that EBV immediate early protein Rta activates autophagy by upregulating ERK activity.^20^ We investigated the role of ERK signaling pathway in Akata and Mutu-I cells exposed to EBV activators with or without the MEK inhibitor U0126 for 24 h. Western blot analysis of phosphorylated ERK1/2, LC3-II and BZLF1 levels revealed that the expression of EBV lytic antigens activated ERK pathway, and that ERK phosphorylation was inhibited by U0126 (Supplementary Figure 1). Moreover, U0126 diminished the amount of LC3-II while delayed and markedly reduced the expression of BZLF1, indicating that ERK signaling affects both autophagy as well as the EBV lytic antigen expression.

We next detected the subcellular distribution of EBV early antigens (EA) and autophagosomes upon lytic cycle induction with or without Bafilomycin A1. For this purpose, we used latently infected Raji cells that, when treated with P(BU)_2_, sodium butyrate and TGF*β*, allow EBV reactivation while undergoing morphological changes, which promote adhesion to the glass. Under these conditions, we visualized endogenous LC3 puncta corresponding to autophagosomes and EBV lytic proteins with antibodies specific for LC3 and for EBV EA. As illustrated in the upper panels of Figure 3b, after 24 h incubation, fluorescence signals enlightened LC3 puncta in the cytoplasm and EBV EA in the nucleus of ~40% of the cell population.^21^ Surprisingly, as shown in the merge of the two images and in the magnified insert, cells that did stain for EBV EA did not stain for LC3 and vice versa, indicating that EBV EA expression blocked autophagy. Addition of Bafilomycin A1 during EBV lytic cycle induction increased the number of LC3 puncta per cell and augmented by ~2-fold, the number of EA-positive cells (Figure 3b, lower panels), confirming the increment of lytic antigens expression observed by immunoblot analysis.

### Bafilomycin A1 increases EBV EA transcription and EBV replication

**Figure 4:**
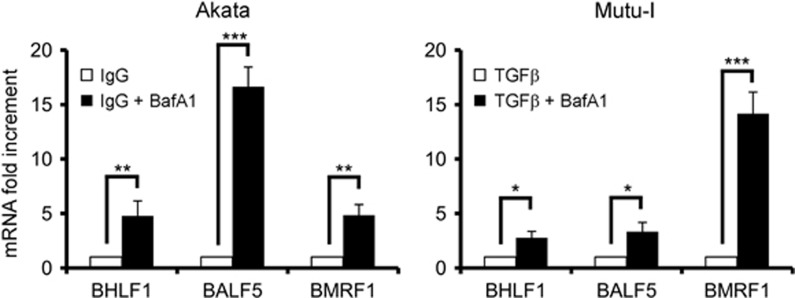
Effects of autophagy inhibition on EBV lytic transcripts. EBV lytic cycle was induced in Akata and Mutu-I cells in the absence or in the presence of Bafilomycin A1. After 24 h, samples were collected and RNA extracted. After retro-transcription, the cDNA was analyzed by real-time PCR with specific primers for BHLF1, BALF5 and BMRF1, and the values were normalized for GAPDH. Fold changes in gene expression levels upon EBV lytic cycle induction were calculated by comparison of the gene expression in Bafilomycin A1-treated cells with the untreated ones, which were assigned an arbitrary value of 1. All reactions were performed in triplicate. ^∗^*P*<0.05, ^∗∗^*P*<0.01 and ^∗∗∗^*P*<0.001

To elucidate whether the Bafilomycin A1-mediated increment of EBV EA was regulated at the transcriptional level, we measured, by quantitative RT-PCR, the transcripts of BALF5, the catalytic component of the viral DNA polymerase, BMRF1, the polymerase accessory protein, and BHLF1, the essential OriLyt promoter.^22^ As shown in Figure 4, exposure of both Akata and Mutu-I cells to Bafilomycin A1 during EBV activation significantly increased EBV lytic gene transcription. The extent of the upregulation of the three EBV transcripts, ranging from ~2.5- to 15-fold, was different in the two cell lines, likely reflecting the different time course of EBV lytic gene expression.

**Figure 5:**
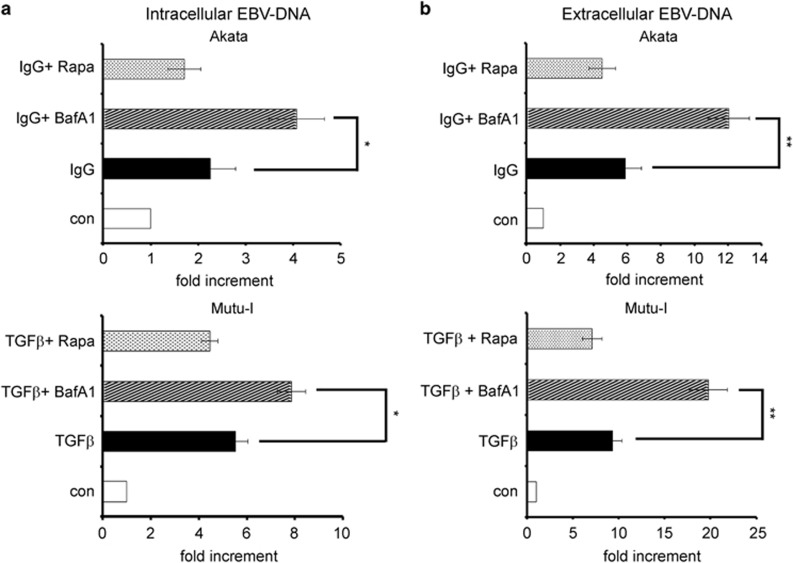
Inhibition of autophagy by Bafilomycin A1 enhances EBV replication. Akata were exposed to IgG for 24 h and Mutu-I cells to TGF*β* for 48 h in the absence or in the presence of Bafilomycin A1 or Rapamycin. Intracellular (**a**) and extracellular (**b**) EBV DNA copies were detected by real-time PCR as described in the Materials and Methods section. The data representing the mean±S.D. of three independent experiments are expressed as fold increment of the treated versus untreated (con) cells. ^∗^*P*<0.05 and ^∗∗^*P*<0.01

To assess whether the enhancement of EBV lytic antigens transcription correlated with increased EBV replication, we determined by real-time PCR that intracellular viral DNA as well as the viral progeny yield released in the medium. Figure 5a shows that intracellular viral DNA copies in Akata cells exposed for 24 h to IgG with Bafilomycin A1 were about twice as many as those measured without. Similarly, treatment of Mutu-I cells with TGF*β* plus Bafilomycin A1 for 48 h increased EBV DNA copies by ~30%. In addition, the results illustrated in Figure 5b show that EBV particles detected in the culture medium of Akata and Mutu-I cells, exposed to EBV activators plus Bafilomycin A1, were about twice as abundant as those found in the medium of control cells. Moreover, in agreement with the results obtained by western blot analysis, in both cell lines, Rapamycin only slightly reduced intracellular EBV DNA copies and released viral particles as compared with the values determined in the cells exposed to IgG or to TGF*β* alone (Figures 5a and b).

### Knockdown of endogenous Beclin1 increases EBV transcription and replication

**Figure 6:**
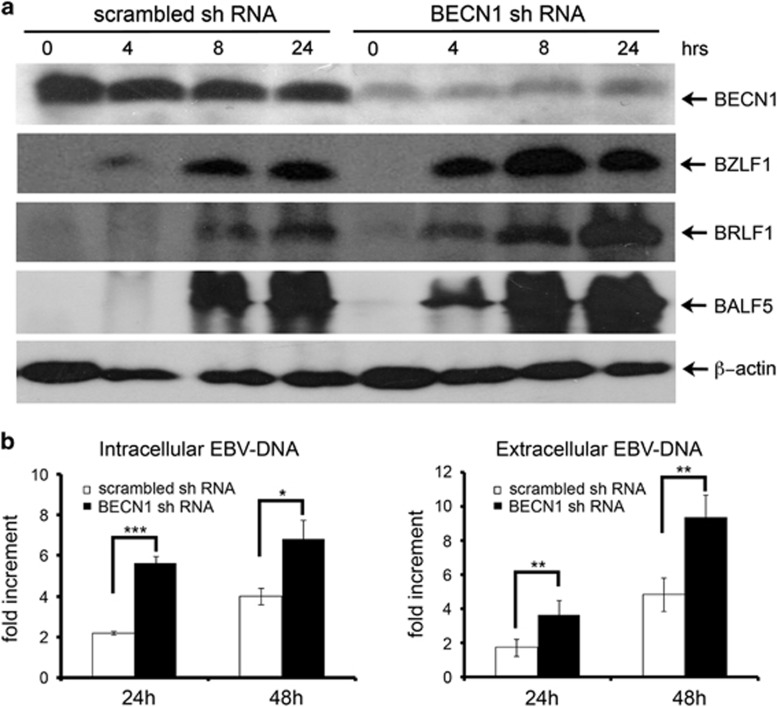
Inhibition of autophagy by Beclin1 knockdown enhances EBV replication. (**a**) EBV-positive Akata cells transfected with shRNAs targeting Beclin1 (BECN1 shRNA) or with scrambled shRNAs were incubated with IgG, and collected at the indicated times. The silencing efficiency of BECN1 shRNA and expression levels of EBV lytic protein BZLF1, BRLF1 and BALF5 were analyzed by immunoblotting. One of three independent experiments is shown. (**b**) Cells were treated with IgG for 24 and 48 h. Both intracellular and extracellular EBV DNA copies were detected by real-time PCR as described in the Materials and Methods section. The data representing the mean±S.D. of three independent experiments are expressed as fold increment relative to time 0. *P<0.05, **P<0.01 and ***P<0.001

To further elucidate the effects of restricting autophagy on EBV lytic infection, shRNA molecular silencing was used to suppress the expression of Beclin1, an essential protein involved in the early steps of the autophagic process. Figure 6a shows that Beclin1-silenced Akata cells exhibited very low levels of the protein as compared with control cells transfected with scrambled shRNA sequences. Notably, upon EBV activation, Beclin1-silenced Akata showed a strong increment in the levels of EBV lytic antigens BZLF1, BRLF1 and BALF5, as compared with control cells. RT-PCR experiments revealed that Beclin1 inhibition enhanced the transcription of EBV lytic genes (data not shown). Moreover, as illustrated in Figure 6b, Beclin1 knockdown in the cells exposed to IgG for 24 h, increased viral DNA replication by ~3-fold and the viral progeny yield by ~2-fold.

Similarly, Beclin1 knockdown in Mutu-I cells determined a significant increment of EBV lytic antigens, intracellular viral DNA and viral particles released in the medium (Supplementary Figure 2).

All together, these results clearly indicate that impairment of the autophagic pathway, also at an early step of the process, highly improves EBV gene expression and replication.

## Discussion

Viruses have been found to increase or block autophagy to enhance their replication. On the other side, several studies indicate that autophagy is activated upon viral infection to hamper viral replication and thereby protect the cells. In general, herpes viruses after an initial stimulation are able to block the autophagic process. HSV1 and HCMV cause an early induction of autophagy in human fibroblasts.^23^ However, at later times during infection, ICP34.5 and TRS1 proteins produced by HSV1 and HCMV, respectively, actively counteract autophagosome biogenesis by binding and inhibiting Beclin1.^9, 11^ In KSHV, the replication and transcription activator RTA is able to enhance autophagy.^24^ Nevertheless, KSHV proteins K7, vBcl2 and vFLIP inhibit various steps of the autophagic pathway.^12, 25, 26^

In this study, we examined the role of autophagy upon EBV activation in three BL cell lines, two of them allowing the virus to execute the complete lytic program and one that contains a defective EBV genome expressing only the immediate early and the early genes. By treating Akata and Mutu-I cells with IgG and TGF*β*, compounds that mimic physiologically relevant mechanisms triggering the transition from latent to lytic infection,^27^ we found the autophagosomal marker LC3-II to increase at the early times, but decrease thereafter, in correlation with a larger expression of EBV early lytic genes. We also observed an increment of LC3-II levels in EBV-negative Akata cells exposed to IgG and in untreated Mutu-I cells during the first 8-h incubation. These data indicate in one case that IgG cross-linking, *per se*, activates autophagy, and in the other case that the autophagic machinery is extremely sensitive and might be perturbed by the experimental procedures (centrifugation and concentration) the cells are subjected to. However, the larger reduction of LC3-II in the EBV-infected Akata and in Mutu-I cells exposed to TGF*β* for 24 or 48 h strongly suggests that EBV lytic antigens expression more effectively contributes to downregulate autophagy. Interestingly, the levels of the autophagic proteins on the average were higher in the uninfected than in the infected Akata cells, possibly indicating a lower basal autophagic activity in the cells harboring the virus. Measurements of the autophagic flux by LC3-II turnover revealed that increased LC3-II levels were due to an increment in the autophagic activity rather than to a block in downstream factors affecting LC3-II turnover, and that this increment occurred at the early times of EBV activation. Moreover, ERK signaling pathway was involved in upregulating autophagy as well as BZLF1 expression.

It was recently reported that EBV immediate early product Rta induces autophagy, and that ATG5 mediates the Rta-induced autophagic activity in transfected 293 T cells.^20^ We did not detect significant variations in the levels of either ATG 5/12 complex or Beclin1 in our EBV reactivation models. It is possible that the full expression of EBV lytic antigens in Akata and Mutu-I allows one or more viral products to effectively counteract the cellular autophagic response. Strikingly in Raji cells that host a deleted EBV genome, we discovered an inverse relationship between EBV EA expression and autophagosomes formation. Because in these cells EBV late antigens are not expressed, it is conceivable to find within the set of EBV EA the effectors of autophagy suppression.

Wortmannin and 3-MA are pharmacological autophagy inhibitors used to block the formation of autophagosomes. However, both compounds are inhibitors of PI3K, the principal mediator of EBV reactivation induced by BCR signaling.^28, 29^ Moreover, PI3K activation is required for Rta-induced activation of *BZLF1* and the early gene *BMRF1*.^30^ Therefore, to avoid compounds that might affect EBV reactivation, we used Bafilomycin A1 and shRNA-based depletion of Beclin1, an essential protein for autophagy initiation to block, respectively, a late and an early step of the degradation pathway. We show here that induction of EBV lytic cycle in Akata and Mutu-I cells in the presence of Bafilomycin A1 or after silencing Beclin1 expression markedly increased the EBV lytic antigens expression, intracellular EBV DNA copies and viral particles release. Recently, siRNA suppression of ATG5 or Beclin1 in EBV-producing cells was shown to reduce EBV lytic gene expression.^31^ These results are contrary to our findings. Possible explanations for this discrepancy might be found in the different strategy applied in order to silence the autophagy genes (siRNA versus shRNA), the time frame of the experiments or cell type specificity. Our data also indicate that Rapamycin, the main activator of autophagy, only slightly reduced EBV lytic genes expression and replication in Akata and Mutu-I cells, suggesting that the virus-mediated inhibition of autophagy is independent of mTOR signaling and occurs in the late steps of the process. Immunofluorescence studies revealed that Raji cells exposed to EBV lytic cycle activators and Bafilomycin A1 almost doubled the percentage of cells in which EBV was activated. At present, we do not know whether the increment of EBV lytic genes expression and replication detected in Bafilomycin A1-treated cells occurs at the single-cell level or depends on a higher number of cells carrying the activated virus. Possibly, both conditions arise at the same time. In fact, inhibition of autophagy might prolong the activity of EBV trans-activators, thereby enhancing EBV lytic gene transcription as we have shown here, and/or increase the number of cells in which the virus is able to complete the lytic gene cascade.

Granato *et al.*^31^ reported that the autophagic activation is instrumental for the early phases of EBV replication, but that in the late steps, the virus blocks autophagy to prevent lysosomal degradation. More recently, Nowag *et al.*^32^ showed that autophagic membranes are stabilized by lytic EBV replication and found LC3-II in the envelope of the viral particles, these results suggesting EBV recruitment of autophagosomal membranes during productive infection. We show that upon activation of EBV lytic cycle, exposure of Akata and Mutu-I cells to Bafilomicin A1 leads to increase in the LC3-II component in the first 8 and 4 h, respectively. Because a prolonged exposure of the cells to the drug mainly impairs autophagosomes–lysosomes fusion,^33^ it is conceivable that the increment of EBV lytic genes expression and replication observed at later times is related to accumulation/stabilization of the autophagic vesicles. In this respect, our data are in agreement with the above reported findings as Bafilomycin A1 treatment would enhance EBV particles production by boosting a mechanism the virus seems to adopt to exploit the cellular autophagic pathway.

In summary, using cellular models in which EBV–host cell interactions are likely to resemble those occurring *in vivo* upon the virus switch from the latent to the productive cycle, we found that autophagy is stimulated in the early phases of EBV lytic reactivation, while it is suppressed later on. We propose that EBV reactivation transiently stimulates autophagy, but that early in the productive cycle of infection, the virus is able to counteract this host defense pathway. The viral products and the molecular mechanisms that allow EBV to suppress autophagy remain to be elucidated. Because Beclin1 silencing increases EBV gene expression and replication, stimulation of autophagy appears to represent the cellular response to the synthesis of the viral products rather than being activated by the virus to favor the early phases of lytic infection.

We report here for the first time that inhibition of autophagy at an early or at a late step of the process highly upregulated EBV gene expression and replication and increased virion yield.

Efficient induction of EBV lytic program in EBV-related malignancies by reagents such as 5 Aza or HDAC inhibitors has been proposed as a kind of oncolytic therapy to kill the latently infected tumor cells, especially in combination with antiviral drugs.^34^ Several clinical trials designed to block autophagy by hydroxychloroquine in multiple cancers are currently carried on.^35^ We envisage that our findings might have clinical relevance, as targeting autophagy with pharmacological inhibitors might be beneficial to maximize the efficacy of an oncolytic viral therapy devised for the treatment of EBV-related cancers.

## Materials and Methods

### Cell lines, EBV lytic cycle induction and reagents

EBV-positive Akata, Mutu-I, Raji and EBV-negative Akata^36^ are BL-derived cell lines. Cells were cultured in RPMI-1640 medium containing 10% fetal calf serum (FCS; Biological Industries) and antibiotics in a 5% CO_2_ atmosphere and maintained at a density of 3.5 × 10^5^/ml. To induce EBV lytic cycle, Akata and Mutu-I cells were diluted to 10^6^/ml in RPMI-1640 medium 10% FCS and treated with 10 *μ*g/ml of goat anti-human IgG (Sigma, St Louis, MO, USA) or 5 ng/ml of TGF*β*1 (BD Pharmingen), respectively. To activate the virus in Raji cells, the culture at a density of 5 × 10^5^ cells/ml in RPMI 1640/2,5% FCS was exposed to 0,04 ng/ml TGF-*β*2 (Genzyme), 2 mM sodium butyrate and 20 ng/ml P(BU)_2_ (Sigma-Aldrich, Milan, Italy). The efficiency of EBV lytic cycle induction was evaluated in all cell lines by counting EA-positive cells after immunofluorescence staining.

Rapamycin and Bafilomycin A1 were purchased from Sigma-Aldrich and were used at a concentration of 5 and 2 nM, respectively.

To evaluate the autophagic flux, cells were incubated with EBV lytic cycle activators in the absence or in the presence of 10 nM Bafilomycin A1 that was added, for each time point, 2 h before collecting the cells. U0126 was purchased from Calbiochem (Milan, Italy).

### Immunoblotting

Equal amounts of proteins (20 *μ*g), as determined by Bio-Rad Protein Assay (Bio-Rad, Hercules, CA, USA), were resolved on a 8% or on a 12% SDS-PAGE and electrotransferred to Amersham Hybond PVDF membrane (GE Healthcare, Milan, Italy). Membranes were then blocked for 1 h at 25 °C in PBS containing 5% nonfat dry milk and 0.1% Tween-20 (Sigma-Aldrich), and were incubated for 1 h at 25 °C or overnight at 4 °C, with the following primary antibodies: BRLF1 and BZLF1(1 : 200 and 1 : 100, respectively, both obtained from Argene Biosoft, Verniolle, France), BALF5 (1 : 100), LC3 (1 : 8000, L7543) and *β*-actin (1 : 5000) were purchased from Sigma, Beclin1(1 : 500; sc-10086) and ATG5 (1 : 200, sc-133158) purchased from Santa Cruz (DBA, Milan, Italy), and phospho-p70 S6 kinase (1 : 1000, 07-018-I Millipore, Merk Spa, Vimodrone, MI, Italy). Phospho ERK1/2 and ERK1/2 antibodies (1 : 500) were obtained from Cell Signaling (EuroClone, Milan, Italy). The membranes were then incubated with the appropriate secondary antibodies conjugated to HRP (1 : 7500, Bio-Rad). The specific signals, visualized by Amersham ECL Plus kit (GE Healthcare) were quantified by densitometric analysis by ImageJ free-share software (http://imagej.nih.gov/ij).

### Fluorescence microscopy

Raji cells were incubated with EBV lytic cycle-inducing agents in the absence or in the presence of Bafilomycin A1 on glass coverslips in a 12-well plate. After 24 h, cells were washed with PBS and fixed with methanol/acetone (1 : 1) for 5 min at −20 °C. Fixed cells were incubated with LC3 antibodies for 1 h at 37 °C, rinsed with PBS and then incubated with anti-rabbit TRITC-conjugated secondary antibodies (Bio-Rad) for 30 min at room temperature. Next, cells were rinsed and incubated for 1 h at 37 °C with FITC-conjugated EBV EA antibodies. After extensive wash, the glass coverslips were mounted onto the slides with 0.1% (w/v) p-phenylenediamine in 10% (v/v) PBS, 90% (v/v) glycerol, pH 8, and the specimens observed by using a Leica DM4000 fluorescence microscope equipped with an FX 340 digital camera (Leica Microsystems, Milan, Italy).

### Total RNA extraction and real-time RT-PCR

Total RNA extracted from 5 × 10^6^ by using Trizol reagent (Life Technologies Italia, Monza MB, Italy) according to the manufacturer's instruction was treated with DNase I (Invitrogen, VWR International, Milan, Italy) for 30 min at 37 °C. DNase was then inactivated by adding EDTA to a final concentration of 5 mM, followed by 10 min heat inactivation at 65 °C. An amount of 1 *μ*g of RNA was reverse transcribed using Super Script III (Life Technologies Italia) and oligo dT. cDNA was then analyzed by real-time PCR using a SYBR green PCR Master Mix (Applied Biosystems, Waltham, MA, USA) on an Applied Biosystems 7900HT Fast Real-Time PCR System based on the manufacturer's specified parameters. Relative RT-PCR was determined using ΔΔCT methods relative to control samples and to the stably expressed housekeeping gene *GAPDH*. The primer sequences used to measure the transcription of EBV lytic genes *BALF5*, *BHLF1*, *BMRF1*, *BZLF1* and *BRLF1* are listed in Supplementary Table 1.

### Quantification of viral DNA

Intracellular viral DNA was extracted using a Wizard genomic DNA purification kit (Promega, Milan, Italy) and treated with RNase A for 30 min at 37 °C. The viral DNA was subjected to real-time PCR using SYBR Green PCR Master Mix (Applied Biosystems) on an Applied Biosystems 7900HT Fast Real-Time PCR System according to the manufacturer's specified parameters. Relative amounts were obtained as above described.

Viral DNA in the supernatant was extracted as described.^37^ Briefly, virus-containing media were treated with 5 U/50 *μ*l of DNaseI (New England Biolabs) for 1 h at 37 °C. After DNaseI heat inactivation (10 min at 70 °C), supernatants were mixed (1 : 1) with lysis buffer (0.1 mg of proteinase K/ml in water) and incubated for 60 min at 50 °C. After a further treatment at 75 °C for 20 min, the released viral DNA was measured by real-time PCR analysis, using a standard curve derived from a serial dilutions of Namalwa cell lysate, which contain two copies of integrated EBV genome per Namalwa cell. Intracellular and extracellular EBV DNA was detected using specific primers for the OriLyt region: 5′-CGTCTTACTGCCCAGCCTACTC-3′ (OriLyt-fwd), 5′-AGTGGGAGGGCAGGAAATG-3′ (OriLyt-rev).

### Lentivirus generation and Akata cells infection

Lentiviral particles were produced by transfecting Hek293T cells with 10 *μ*g of the lentiviral shRNA vectors pLKO Scramble (Addgene, Cambridge, MA, USA) or pLKO Beclin1 (TRCN0000033550, Sigma-Aldrich), together with the packaging vectors pMD2.G (2.5 *μ*g, Addgene) and psPAX2 (7.5 *μ*g, Addgene). Forty-eight hours later, the supernatants containing the lentiviral particles were collected, supplemented with polybrene (4 *μ*g/ml) and FCS (10%), filtered (PES 0.45 *μ*M filters, Corning) to remove cell debris and incubated with Akata cells for 8 h. Mutu-I cells were infected with lentivirus by mixing cells with the culture supernatant in the presence of 8 *μ*g/ml polybrene and then centrifuging the mixture at 450 × *g* for 90 min. Infected cells were analyzed starting from 48 h after infection. Stable cell lines expressing siRNAs were selected by supplementing the culture medium with 1 or 2.5 *μ*g/ml puromycin for Akata or Muti-I cells, respectively.

### Statistical analysis

All data were analyzed using GraphPad Prism6 (GraphPad Software, Inc., La Jolla, CA, USA). The data, expressed as the mean±S.D., were analyzed by two-way ANOVAs to determine statistical differences. A *P*-value of <0.05 was considered statistically significant.

## Abbreviations

EBV: Epstein Barr virus
BL: Burkitt's lymphoma
anti-IgG, IgG: anti-immunoglobulin G
HDAC: histone deacetylase
TGF*β*: transforming growth factor beta
ATG: autophagy-related gene
mTOR: mammalian target of rapamycin
BECN1: Beclin1
PI3K: phosphatidylinositol 3-kinase
LC3: microtubule-associated protein light chain 3
HSV1: herpes simplex virus type 1
HCMV: human cytomegalovirus
KSHV: Kaposi's sarcoma-associated herpesvirus
HIV: human immunodeficiency virus
