# Supplementary material for: Inhibition of autophagy in EBV-positive Burkitt's lymphoma cells enhances EBV lytic genes expression and replication
Source: Cell Death Dis. 2015 Sep 3;6(9):e1876–. doi: 10.1038/cddis.2015.156 (PMC4650432; doi:10.1038/cddis.2015.156)
Supplement: Supplementary Figure Legends [file cddis2015156x1.doc]

**Legends to Supplementary Figures**

**Supplementary Figure 1. EBV activation affects autophagy via ERK signaling.**

Akata cells and Mutu I cells were treated with IgG or TGFβ, respectively, in the absence or in the presence of the ERK inhibitor U0126 at 1µM. The cells were harvested at the indicated times and the lysates analyzed by immunoblotting with specific antibodies for the indicated proteins.

**Supplementary Figure 2. Beclin1 knockdown in Mutu I cells enhances EBV replication.**

(**A**) Cells transfected with shRNAs targeting Beclin1 (BECN1 shRNA) or with scrambled shRNAs were incubated with TGFβ and collected at the indicated times. The silencing efficiency of BECN1 shRNA and expression levels of EBV lytic protein BZLF1 and BALF5 were analyzed by immunoblotting. One of three independent experiments is shown. (**B**) Cells were treated with TGFβ for 24 and 48 hours. Both intracellular and extracellular EBV DNA copies were detected by real-time PCR as described in the Methods. The data representing the mean ± SD of 3 independent experiments are expressed as fold increment relative to time 0. ***P* < 0.01; ****P* < 0.001.
