# Supplementary figures and images for: Inhibition of autophagy in EBV-positive Burkitt's lymphoma cells enhances EBV lytic genes expression and replication
Source: Cell Death Dis. 2015 Sep 3;6(9):e1876–. doi: 10.1038/cddis.2015.156 (PMC4650432; doi:10.1038/cddis.2015.156)

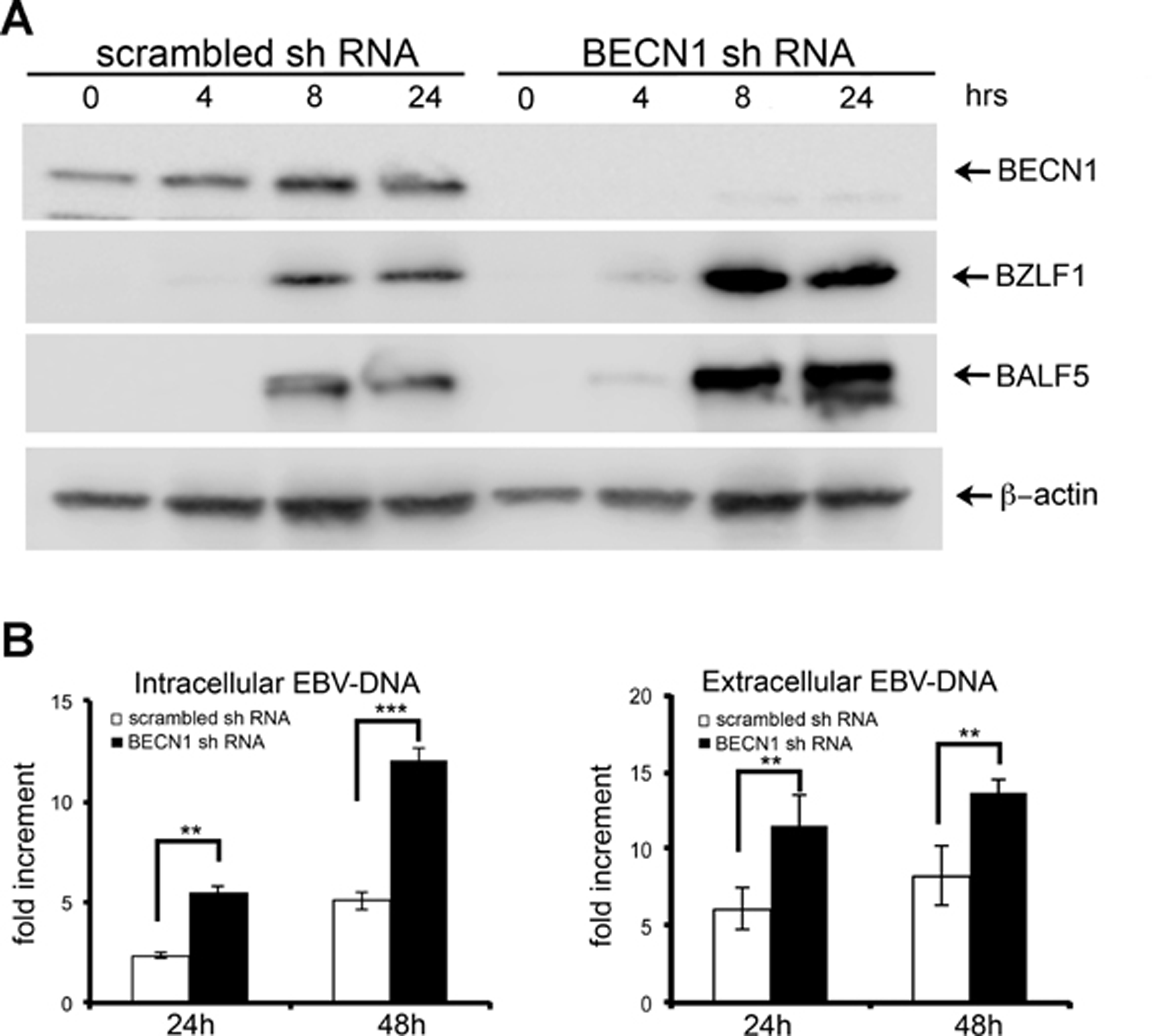

Supplement: Supplementary Figure 1 [file cddis2015156x2.tif]

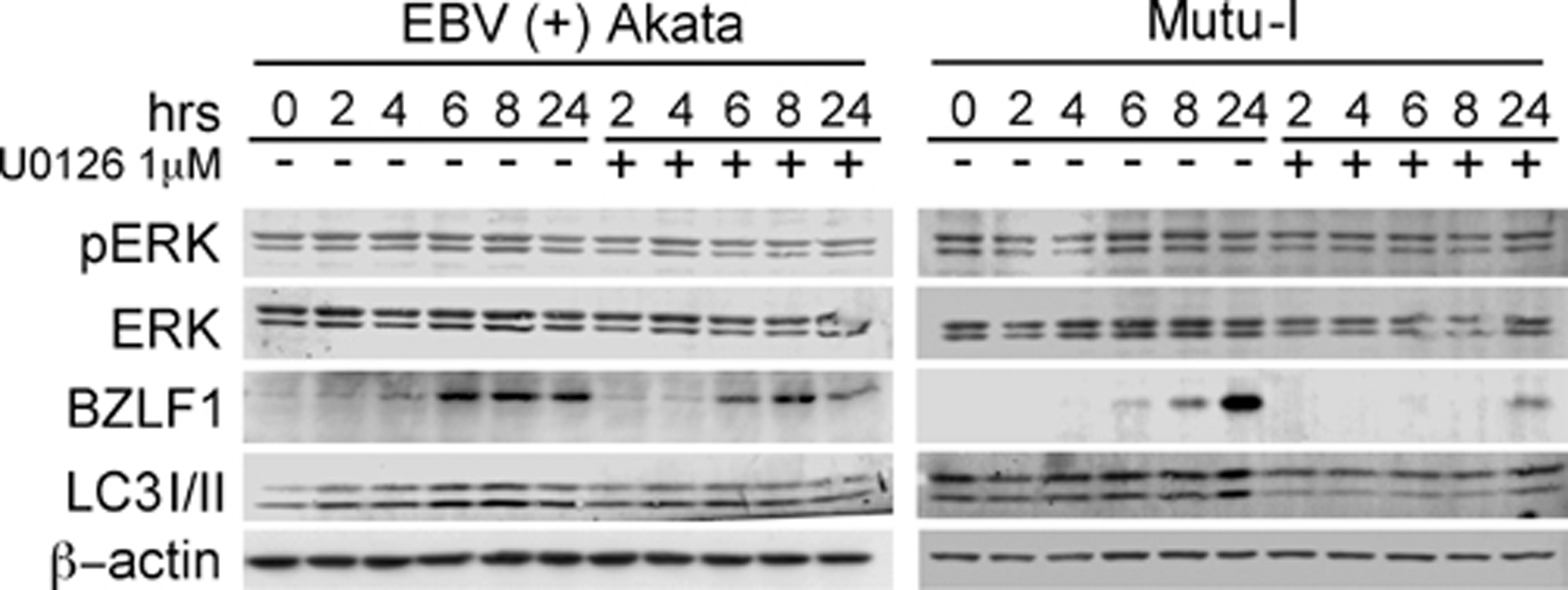

Supplement: Supplementary Figure 2 [file cddis2015156x3.tif]
