# Supplementary material for: Inhibition of autophagy in EBV-positive Burkitt's lymphoma cells enhances EBV lytic genes expression and replication
Source: Cell Death Dis. 2015 Sep 3;6(9):e1876–. doi: 10.1038/cddis.2015.156 (PMC4650432; doi:10.1038/cddis.2015.156)
Supplement: Supplementary Table 1 [file cddis2015156x4.doc]

**Table 1. Primers sequences used for RT-PCR**

BALF5 fwd CTG TGA GGT GGG CGA CCT C

BALF5 rev CCG ATG GGA ACT CGT AGA CC

BHLF1 fwd CGGGTTGGTGGCACTGTT

BHLF1 rev GCATGGCGAAGTAGACAGGTTA

BMRF1 fwd TTGGGCAGGTGCTGTTGAT

BMRF1 rev TGCCCACTTCTGCAACGA

BZLF1 fwd TCTGAACTAGAAATAAAGCGATACAAGAA

BZLF1 rev TTGGGCACATCTGCTTCAAC

BRLF1 fwd GAGCGATGAGAGACCCATATTC

BRLF1 rev GAACATACCTTCCCGGCTATC

GAPDH fwd agccacatcgctcagacac

GAPDH rev gcccaatacgaccaaatcc
